# Potential for combining semantics and data analysis in the context of digital twins

**DOI:** 10.1098/rsta.2020.0368

**Published:** 2021-10-04

**Authors:** Birgit Vogel-Heuser, Felix Ocker, Iris Weiß, Robert Mieth, Frederik Mann

**Affiliations:** Institute of Automation and Information Systems, Technical University of Munich, Boltzmannstr. 15, 85748 Garching, Germany

**Keywords:** data analysis, knowledge-based systems, production systems, digital twins

## Abstract

Modern production systems can benefit greatly from integrated and up-to-date digital representations. Their applications range from consistency checks during the design phase to smart manufacturing to maintenance support. Such digital twins not only require data, information and knowledge as inputs but can also be considered integrated models themselves. This paper provides an overview of data, information and knowledge typically available throughout the lifecycle of production systems and the variety of applications driven by data analysis, expert knowledge and knowledge-based systems. On this basis, we describe the potential for combining data analysis and knowledge-based systems in the context of production systems and describe two feasibility studies that demonstrate how knowledge-based systems can be created using data analysis.

This article is part of the theme issue ‘Towards symbiotic autonomous systems’.

## 1. Motivation

Increasingly complex products require increasingly complex and powerful production systems. To create and operate such advanced production systems, highly specialized engineers with various technical back- grounds create lots of engineering information. In order for them to cooperate effectively, their knowledge has to be combined. At the same time, cheap data storage and advances in electronics allow engineers to aggregate immense amounts of data. Due to the long lifecycle of production systems, which may well exceed the career of individual employees [1], it is crucial for companies to manage their data, information and knowledge. Only then can they ensure successful design and operation of production systems.

Novel technologies, such as digital twins (DTs), aim to make this data and information more accessible. To also leverage the available information, technologies such as data analysis and knowledge-based systems (KBSs) become ever more important. With this paper, we aim to provide an overview of current technologies from data analysis and KBSs and provide a perspective on how they can be combined. An overview of available data, information and knowledge and the dependencies among them is the basis for effective cooperation. Hence, this paper will address the research question ‘Which data, information and knowledge is available throughout the lifecycle of production systems?’ (*RQ1*). As usual in engineering, it is important to choose the right tool for a task. Even though novel technologies such as data analysis and KBSs seem promising, we will analyse which use cases throughout the lifecycle of production systems, or even automated production systems (aPSs), can be expected to benefit from data analysis and KBSs (*RQ2*). Based on this analysis, we will also give an overview of approaches from data analysis and KBSs that have already been successfully applied to production systems (*RQ3*). Finally, data analysis and KBSs are two complementary technologies. While data analysis enables a bottom-up approach for creating new insights, KBSs rely on logic to infer new insights in a top-down way. To bridge the gap between these two technologies, we will also address the research question ‘How can data analysis and KBSs be combined in the context of production systems?’ (*RQ4*).

The rest of the paper is structured as follows. Section 2. gives an overview of the data, information and knowledge available throughout the lifecycle of production systems. Additionally, we introduce technologies and frameworks for data analysis and KBSs in the context of production systems. Section 3. shows how the different sources of information can be leveraged, which approaches from data analysis and KBSs may complement one another, and how engineers can integrate them. Subsequently, §4. provides some palpable examples. This paper concludes with a summary and an outlook.

## 2. Related work

This section describes the variety of available data, information and knowledge in smart manufacturing, introduces the notion DT and provides an overview of related work in data analysis and KBS for smart manufacturing.

### (a) Data, information and knowledge in smart manufacturing

As indicated by the notions, data analysis methods leverage data, while KBSs rely on knowledge. The so-called data, information, knowledge, wisdom (DIKW) pyramid [2] makes the difference apparent. For instance, data may simply be a number such as ‘10.0’. Putting data into context results in information, e.g. ‘pressure is 10 bar’. Based on information, knowledge describes the ability to infer new insights, e.g. ‘valve has to be opened to reduce malfunction’. The additional notion of ‘wisdom’, however, is controversial [3]. A distinction of information into the categories function, behavior and structure can be traced back to the seventies [4]. Here, the function is formulated in terms of requirements to be met for achieving a system’s intended purpose. This distinction is also represented in the product design model defined by the VDI/VDE [5]. Information is oftentimes captured using models, which are defined as ‘representation[s] of reality intended for some definite purpose’ [6].

Models are created for one of four purposes. Specifically, they can be descriptive, diagnostic, predictive or prescriptive [7]. While descriptive models describe the world as it is, i.e. ‘what has happened’, diagnostic models aim to explain why something happened. Both predictive and prescriptive models are oriented towards the future, with predictive models trying to answer the question ‘what will happen?’, and prescriptive models aimed to infer appropriate actions. Biffl *et al.* [8] distinguish various types of information captured in models throughout the lifecycle of a production system for different layers, ranging from construction elements to entire production networks, cp. table 1. Note that information relevant for the end-of-life can be reused from the prior phases.

**Table 1.** Selection of information artifacts throughout the lifecycle of a production system adapted from Biffl *et al.* [8].

layer	design	operation and maintenance
production network	requirements, constraints	volume planning, Key Performance Indicators (KPIs), technical rules, order data, quality data
production line (segment)	two-dimensional-layouts	resource allocation, KPIs, logistics data, quality data, maintenance reports, test data
work unit, work station	layout plans, behavior models, three-dimensional-geometry-models, electrical construction, fluid plans, control programs, safety concepts	resource KPIs, resource states and alarm logs, sensor data, order data, production process control data, maintenance report, test data
component	behavior models, CAD construction, control programs, safety concepts, part lists	component state and alarm logs, production process control data, sensor data, test data
construction element	part lists, mechanical/electrical specifications, CAD-based construction	component health information

**Figure 1.**
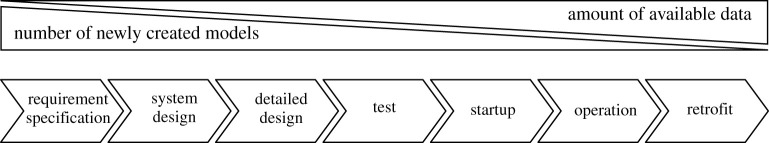
Model and data creation throughout the lifecycle.

When analyzing this data and information available, it becomes apparent that the number of newly created models decreases throughout the lifecycle of a plant, while the amount of available data increases, cp. figure 1.

The tooling landscape for capturing all this heterogeneous information is also highly diverse and includes domain-specific tools. For instance, there are computer-aided design (CAD) tools for mechanics and electrics/electronics, computer-aided engineering (CAE), integrated development environments (IDEs) for software engineering and various modelling tools even for early design phases. These tools are oftentimes proprietary, with dedicated languages and file formats, thus making an exchange of information potentially challenging. Since all this information is related to the same system, there exist many dependencies [9], which need to be managed to successfully design and operate production systems. Additional challenges arise from legacy workflows [10], and management of model dependencies between the different documents throughout the design process is still an active field of research. Among others, approaches have been developed to manage such dependencies using business process model and notation (BPMN) [10] or via formal knowledge representations [11]. The latter uses the notions *information*, *information concretization*, *information carrier*, *actor* and *system* to build a knowledge graph that provides an overview of available information and its dependencies.

‘While appropriately qualified, interested and motivated people could make do with imprecisely expressed informational content, electronic information processing systems absolutely require exact and well-structured definitions’ [12]. Such well-structured definitions can be realized in the form of ontologies, i.e. formalized knowledge bases. Gruber [13] defines an ontology as an ‘explicit specification of a conceptualization’. While an ontology’s terminological component (TBox) describes facts about the notions included, its assertional component (ABox) describes the instance level. Different kinds of ontologies, namely application ontologies, reference ontologies and top-level ontologies, should be distinguished [14]. Application ontologies rely on specific universals and particulars in combination with inference mechanisms for executing some specific task [15]. For instance, there is an ontology dedicated to providing feasibility feedback in early design phases [16]. In contrast, reference ontologies, such as MASON [17], contain application independent knowledge and can be reused by various application ontologies. In comparison to application and reference ontologies, top-level ontologies are the most generic and formalize notions such as *continuant* and *occurrent* [15]. Well-known examples include basic formal ontology (BFO) [15] and Descriptive Ontology for Linguistic and Cognitive Engineering (DOLCE) [18]. Such top-level ontologies allow engineers to integrate and organize information even across domains [15]. This enables engineers to combine ontologies and thus increase semantic interoperability. This results both in an increase in knowledge and its quality [19]. An in-depth overview of top-level ontologies in the context of smart factories is presented in prior work [20].

### (b) Digital twins

In the context of smart manufacturing, DTs can be used to accumulate the information related to products, processes and resources. DTs can help to support various use cases, ranging from smart production to mass personalization [21]. According to Löwen [22], an integrated plant model ‘maintained and kept consistent throughout the entire life of the plant’ is a DT. Such integrated models help to increase reuse and also ensure an early integration. There are various interpretations of the notion DT. On the one hand, academic definitions range from an ‘integrated simulation of a system’ [23] to a ‘dynamic virtual representation of a physical object or system across its lifecycle’ [24] to a high-fidelity representation of the operational dynamics of the corresponding asset, which requires near real-time synchronization between the two [25]. Industry on the other hand mostly emphasizes the economic benefits but also understands a DT as a ‘real-time digital replica of a physical device’ [26] or a ‘virtual representation of a physical asset or system throughout its lifecycle’ [27]. All these definitions have in common that they perceive a DT as a virtual representation of a physical entity, which exists throughout the entire lifecycle. Two major challenges for DTs include their creation from available data and their semantic unambiguousness. The first challenge may be addressed using data analysis methods and existing models. The second challenge requires formalized knowledge representations, as they can be created using semantic web technologies (SWT) [28,29].

### (c) Data analytics for smart manufacturing

Manufacturing with its sensor and actuator data generates vast amounts of data—even more than other sectors [30]. This data provides high potential for data-driven improvements in smart manufacturing. Therefore, prerequisites are big data and machine learning (ML) algorithms [31]. Typical examples for ML use cases in manufacturing are product quality prediction to decrease scrap or condition monitoring to prevent unplanned shutdowns due to equipment failures. The overarching goal is the improvement of the overall equipment effectiveness (OEE), in other words, the increase of the performance and the availability of a machine and improvement of product quality. However, despite the progress in hardware and in the algorithmic processing of such vast amounts of data, real industrial applications are still rare. One of the main reasons is the challenge due to characteristics of the data. Industrial data often represents only positive occurrences since the well-engineered machines run smoothly. Process cycles resulting in bad product quality or sudden, fatal equipment failures are rare or not existent, although they are required for learning [32]. Liu *et al.* [33] refer to this as the representative problem [33]. Consequently, hybrid approaches combining data-driven and model-driven methods to compensate the lack of data are a key research need [32]. Further challenges of industrial big data emphasize the need for hybrid approaches. The challenges given in table 2 can be met by the combination of data analytics and KBSs. Missing meta data, e.g.units of a sensor value, can be acquired by the technical documentation of the sensor. A knowledge base, connecting technical documents and the data base, would increase the usability of data-driven approaches significantly. However, hybrid approaches are still lacking the theoretical foundations and mechanisms to link data-driven and model-driven approaches [39].

**Table 2.** Selected challenges of ML in manufacturing.

challenge	example
information incompleteness [33]/ missing meta data [34]	Missing units for the gathered sensor and actuator data: even though the technical documents of the equipment or sensor include such information, data analysts and the user of data-driven models often suffer from missing meta data. A complete representation of an aPS requires the inclusion of all information (data, expert knowledge, technical documents, etc.).
inconsistency [33]	Changing sampling rates: adjustments of the process, introducing new tasks on the programmable logic controllers (PLCs) or other changes may influence the sampling rate of the data. It is important to detect such inconsistency to prevent wrong interpretation of data-driven results.
causality vs. correlation [35,36]	Correlation ≠ causality: the correlation of two sensors represent statistical probability. A physical or technical dependency can not be proven without considering expert knowledge.
human influence [37,38]	Trust, transparency, explainability: to ensure trust in ML and to cause the human to realize the recommendations, transparency must be given and the results have to be explainable. However, so-called black-box models miss these aspects nowadays.
high dimensionality [34,39,40]	Irrelevant and redundant information: aPSs are equipped with a high number of sensors. However, only a subset of this data is relevant for a specific use case. The irrelevant and redundant information impact the performance of ML and need to be identified.
algorithm selection [34,41]	Overwhelming number of different algorithms: selecting the best algorithms for the use case and the data characteristics is crucial to receive valid results. However, this is a challenging task due to the high number of different algorithms. A knowledge base is required, supporting data analyst and process expert in selecting appropriate algorithms.
interpretation of results [34,37]	Wrong interpretation may lead to wrong decisions: condition monitoring aims to observe changes in the machine behavior in order to take appropriate maintenance action. However, the interpretation if a change of behaviour is critical often remains for the operator.
heterogeneous data sources [39,40]	Link between data sources is missing: sensor and product quality data is processed in different systems. The link between those two sources is required to train a product quality model. However, the production of mass products such as small gear wheels does not allow the link between the data sources since the gear wheels are not traced individually.

### (d) Knowledge-based systems for smart manufacturing

Engineers have discovered KBSs as an appropriate measure to cope with specialization and increasing available knowledge caused by increasing complexity. Hence, a wide variety of KBS-based approaches has been developed for the smart manufacturing domain. Such applications include feasibility feedback for engineers in early design phases [16,42], which requires knowledge regarding both product and resource. Similarly, KBSs are suitable for managing inconsistencies in interdisciplinary design. Such approaches range from combinations of graphical modeling languages and rules [43] to Bayesian reasoning [44]. KBS can even be applied in the context of human resources to manage how employees are allocated to projects, based on their competences [45,46]. In a production context, applications for KBS range from the consistent initialization of agents within a multi agent system (MAS) [47] to matchmaking between required processes and machine skills [48]. Further approaches aim to improve semantic interoperability across the supply chain and to support supplier identification [49] and to support cloud-based business interactions [50]. Finally, KBSs also play an important role in the context of DTs to provide clear semantics, e.g. via web ontology language (OWL) representations of DTs [28,29]. Even though all these approaches seem promising by themselves, they all share several challenges, cp. table 3. Only when these challenges are successfully addressed will KBSs find their way into large-scale industrial applications. For some of these challenges, ML approaches may serve as a solution.

**Table 3.** Selected challenges of KBS in manufacturing. pcc

challenge	example
ontology creation	While the manual creation of the ontologies’ TBoxs is realistic, the population of ontologies, i.e. the creation of the instance level still poses a major challenge for engineers. Ideally, existing sources of information should be reused, but oftentimes the information stems from heterogeneous sources and is not easily interpretable, e.g. when only available as natural language text.
ontology evolution	Ontologies not only need to be created, but they also have to be updated continuously. Compared to ontology creation, information not only has to be formalized, but the existing ontology also needs to be checked for correctness continuously.
scalability	Even though many benefits arise from integrating information, there are also scalability limitations. There may be huge numbers of nodes and edges, if an ontology aims to capture an entire production system. Nodes would be required for everything, from products to employees to machines and their multitude of variables.
knowledge representation	Even though ontologies are well suited to represent the way humans think, namely in nodes connected via edges, they are not suitable for all types of data. For instance, there are limitations to how arithmetic operations can be performed using SWT and Binary Large Objects are also problematic.
understandability	While being computer-interpretable, it may be hard for humans to grasp the information captured by ontologies and insights gained from this information. For instance, even graduate mechanical engineering students have difficulties understanding the explanations provided by reasoners integrated into the ontology editor Protégé.
ontology combination and reuse	While combination of ontologies is in principle supported through the Open World Assumption, ontology matching is still a major challenge in practice. This is, among other reasons, due to the heterogeneity of ontologies in terms of design choices, domain of interest, structure and terminology.

## 3. Towards combining data analysis with knowledge-based systems

This section provides an overview of applications, which would benefit from an integration of data analysis, KBS and expert knowledge. Subsequently, we discuss the information required for these applications and its availability and finally delineate how the three technologies could benefit from an integration.

### (a) Applications

**Figure 2.**
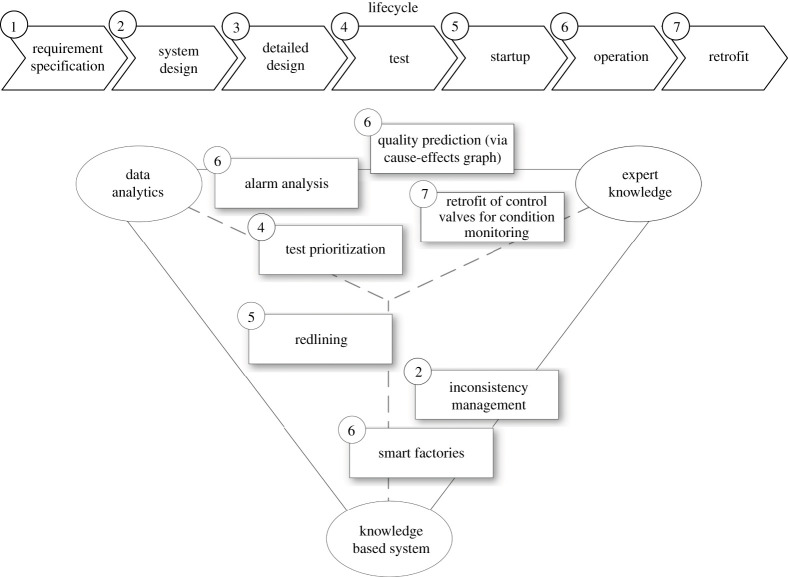
Overview of knowledge-intensive applications throughout the lifecycle of a production system.

The overview of available information shows the diversity along the lifecycle of an aPS. In order to utilize this information, data analytics, expert knowledge and KBS can be used. These use cases reflect the need for a hybrid approach, combining the three aspects to compensate their disadvantages, as also shown in §2.. In the following, particular research applications developed at the Institute of Automation and Information System at the Technical University of Munich are provided, illustrating the possible leverage effect of integrating data analytics, expert knowledge and KBS, cp. figure 2. The application examples originate from a variety of research projects, including SIDAP, IMPROVE, CRC 768 (especially A6, D1 and T5), V&VArtemis and DAVID.

Systems design: interdisciplinary engineering is challenging due to the number of engineers involved, who have different views on the same system. This results in overlapping and potentially contradictory models, which need to be combined to realize a functioning product. Here, not only inconsistencies among the various engineering disciplines, e.g. mechanics, electrics/electronics and software, need to be managed [43] and resolved [51], but feedback regarding feasibility of products with regard to the available resources [16] should also be given as early as possible. These approaches require the expertise of the engineers involved, but can also benefit immensely from formal knowledge representations, which can be integrated. Data analysis may complement these approaches to identify previously unknown correlations.

Test: efficient regression testing of aPSs is highly dependent on the experience of the testing engineers, as they have to select the test cases to be re-run within the limited time carefully based on various test criteria. As the number of test cases increases with the complexity of the aPS, test planning becomes time-consuming and challenging for test engineers. Extracting expert knowledge (e.g. on situational testing strategy) and supplementing it with data-driven approaches (e.g. analysis results of historic test data) would reduce time, effort and increase efficiency [52].

Startup: during startup of an aPS changes to the original design can occur, e.g. different wiring compared to the circuit diagram. Common practice is the handwritten annotation of changes on the circuit diagram, called redlining. To ensure consistency, the changes have to be traced back to the original documents. Data-driven approaches to detect and identify the handwritten annotations automatically could improve this process significantly [53].

Operation: data-driven product quality prediction is an efficient approach to increase the OEE in processes, where the product quality cannot be measured during production or only with disproportionately high effort. However, historic sensor and actuator data of well-designed aPSs often lack data of production cycles leading to bad data quality. Therefore, a solely data-driven prediction approach very likely ends in inadequate results. Expert knowledge about the dependencies between process parameters and product quality can support the feature selection for data-driven modelling, increasing the significance of the prediction models. So-called Cause-Effect Graphs are an appropriate representation of expert knowledge. [54,55]

Operation: alarm systems of aPSs are designed to inform operators of abnormal situations and faults of the system. Since these alarms often propagate through the interdependent equipment, alarmfloods are observed regularly, e.g. low pressure and flow alarm of a pipe will further raise a low level alarm of the attached tank. Data-driven pattern recognition methods are applied to detect alarm sequences in historic alarm data to support operators in case of alarmfloods by identifying the root alarm [56,57]. However, detecting alarm sequences in complex systems with its chattering alarms and overlaying sequences is a challenging task. Therefore, additional information, e.g. from the machine layout, should be analysed to improve data-driven methods for alarm analysis [58].

Operation: smart factories can be realized using a combination of intelligent resourcess [59] and products [60]. Such intelligent agents require knowledge bases (KBs) that allow them to make decisions, infer new insights and communicate in an unambiguous way. KBSs provide the means to achieve appropriately formalized and compatible KBs [47]. In order to continuously evolve these KBs, intelligent agents within smart factories would also benefit from advanced data analysis, e.g. to continuously adapt their objective functions.

Retrofit: data-driven methods require the availability of data representing the underlying aPS. However, providing an aPS with all possible sensors in maximum precision is not economical. Therefore, a reasonable selection of sensors measuring the process has to be made. For condition monitoring in control valves expert knowledge provides the required information for a retrofit. Based on a failure mode and effect analysis, a team of experts (process experts and data analysts) evaluated which sensor signals are suitable to indicate specific fault mechanisms [61]. Based on the retrofitted valves, efficient data-driven condition monitoring can be developed.

### (b) Overview of available and required information

The variety of applications shown in figure 2 requires very heterogeneous data, information and knowledge created throughout the entire lifecycle of production systems. At the same time, the available inputs differ greatly in structure, formalization and interpretability.

Table 4 provides an overview of the information required for the applications delineated and its availability. It illustrates that all applications require data, information and knowledge from various sources. Hence, the combination of the technologies data analysis and KBS complemented with expert knowledge seems promising for all applications.

**Table 4.** Available and required information for various applications throughout the lifecycle of production systems.

application	required information	availability
inconsistency management	domain-specific models, product specifications, description of resource capabilities, mappings of the models or rules for inconsistency detection	models from engineering, expert knowledge, engineering documents for product specification, DTs for capabilities, reference ontologies and ML approaches (ideally) or expert knowledge (more realistic) for matching
test prioritization	historic test data, metrics to measure test results	documentation from prior projects, standards, gut feeling of testers
redlining	ECAD models and documents with on-site changes	models from engineering, changes made by operators (usually handwritten)
quality prediction	sensor data, quality data, algorithms	matching difficult due to time shift, quality can only be assessed via destructive testing
alarm analysis	alarm data, algorithms to be used for analysis	alarm data available from the field level, algorithm choice requires expert knowledge
smart factories	product and resource status, product features to be realized, resource capabilities, objective functions	product specification, specifications or DTs of the resources, expert knowledge and possibly customer preferences for defining objective functions
retrofit of control valves	failure mechanisms, physical dependencies	expert knowledge distributed across several employees or even companies along the supply chain

### (c) Synthesis

In order to support the engineering applications and meet their needs regarding data, information and knowledge, cp. table 4, the challenges regarding data analysis, cp. table 2, and KBS, cp. table 3, need to be addressed. Table 5 delineates how these two technologies may support each other.

**Table 5.** Selected challenges and potential solutions by leveraging data analytics and KBS in an integrated way (DA = data analysis, KBS = knowledge-based systems).

challenge	origin	potential approach
incomplete information	DA	KBS may be applied to infer missing information, e.g. missing units based on the associated sensor class.
inconsistency	DA	Inconsistencies in the data set may be identified and possibly resolved using SWT during preprocessing.
missing causality	DA and KBS	KBS may be used to formalize causalities experts are aware of, which can then be used to assess analysis results. In parallel, DA should be applied to identify potential causalities, which may be formalized in KBS if confirmed by experts.
interpretation of results	DA and KBS	If formalized KBs are available, inference mechanisms may be applied to infer unambiguous results. However, there are still limitations to the understandability of explanations provided by reasoners.
high dimensionality	DA	If experts can specify relevant features, their knowledge may be incorporated in a KB similar to causalities. These KBs can then be leveraged in a preprocessing step.
algorithm selection	DA	In order to support engineers in selecting appropriate algorithms, support systems relying on KBS seem promising.
heterogeneous data sources	DA and KBS	While DA may help to identify similarities in heterogeneous data sources, alignment with a common ontology for different data sources would enable semantic integration.
ontology creation	KBS	DA may be applied to populate the ontologies’ ABoxs. For instance, text mining allows engineers to automatically extract information from textual engineering documents.
scalability	KBS	DA could be applied to identify the views typically used by engineers from their interaction with KBS.
ontology combination	KBS	ML techniques may be applied to support ontology matching approaches. For instance, Natural Language Processing may be applied for terminological analyses as a part of the matching process.

Table 5 shows that data analysis can serve as a suitable technique to turn data, i.e. hidden information, into explicit knowledge representations. That way, the manual effort for creating KBS seems more feasible, thus addressing one of the key challenges for the application of KBS in the context of production systems.

In order to transform data into information, there are three core approaches. First, engineers could apply data analysis to identify correlations, which can then be formalized. Additionally, optical character recognition (OCR) techniques allow engineers to interpret the data, e.g. included in scans of circuit diagrams. Second, interviews may serve as a way to extract and subsequently formalize the knowledge of experts. Even though highly valuable, this second approach requires much manual effort and psychological effects may have to be considered. Third, engineers can apply natural language processing (NLP) [62] to analyse the content of textual documents. In a subsequent step, technologies such as model transformations and ontology-oriented programming [63] can be applied to turn information into knowledge. Using these technologies, information can be formalized that is captured in structured documents, such as spreadsheets [42,47], or in models [64].

## 4. Feasibility studies

In the following, we delineate how ontologies can be created by leveraging metadata gained from crawling file systems and dependencies identified based on text mining of forums.

### (a) Ontology population leveraging a file system crawler

The ontology presented by Ocker *et al.* [11] can serve as a basis to represent available information and can thus be used to configure DTs. Among other applications, this ontology can be applied for finding specific information *(C1)*, analyzing dependencies *(C2)*, checking compatibility between available models and tools *(C3)* and tracking the propagation of changes made to models *(C4)*. Respective queries are available implemented in SPARQL protocol and RDF query languages (SPARQLs). For these applications, however, the available documents, models and data sources have to be described in the ontology’s ABox which, if done manually, would require enormous efforts. One step towards automating the ontology’s population is the use of a file system crawler that analyses metadata of available documents.

In a first step, we created an overview of the variety of engineering tools and the file formats engineers commonly use. Table 6 shows an excerpt of drawing file types created using mechanical CAD tools. We created analogous classifications for electrical/electronic CAD tools, e.g. Zuken’s E3, IDEs, e.g. VS Code and CODESYS, and modeling tools, such as the Eclipse Modeling Framework. Overall, we analysed 20 tools and linked them with more than 200 file formats that can be identified via unique file extensions. As indicated by table 6, these file formats can be linked to the kind of information they contain, e.g. technical drawings from the mechanics perspective. For this linking step, we relied on the overview of data and information available throughout the lifecycle of a production system, cp. §2. and 3.. To improve interpretability of the files’ contents, we classified them as either behavioural or structural information and also distinguished descriptive, diagnostic, predictive and prescriptive models. So far, functional information was not considered, as tools for requirements management were not within the scope of this analysis. This could be added analogously, though.

**Table 6.** Excerpt of file extensions used by selected mechanical CAD tools for technical drawings.

tool	supplier	file extension
AutoCAD 2021	Autodesk	.dwg
Inventor 2021	Autodesk	.idw
CATIA V5	Dassault Systèmes	.CATdrawing
Solid Works	Dassault Systèmes	.slddrw
Creo 7.0	PTC	.drw

For an automated analysis of the files available within a file system, we implemented a file system crawler using Python. Starting from a path specified by the user, the crawler recursively checks all subfolders and files contained therein, cp. algorithm 1. Based on the file format, we infer what kind of information is contained in a file and store additional metadata such as timestamps. If available, e.g. in the case of PDF or HTML files, further metadata such as the author can also be extracted. This information is used to populate the ontology’s [11] ABox using Owlready2 [63] and is available on GitHub as a fork^1^ from the original information model project.^2^


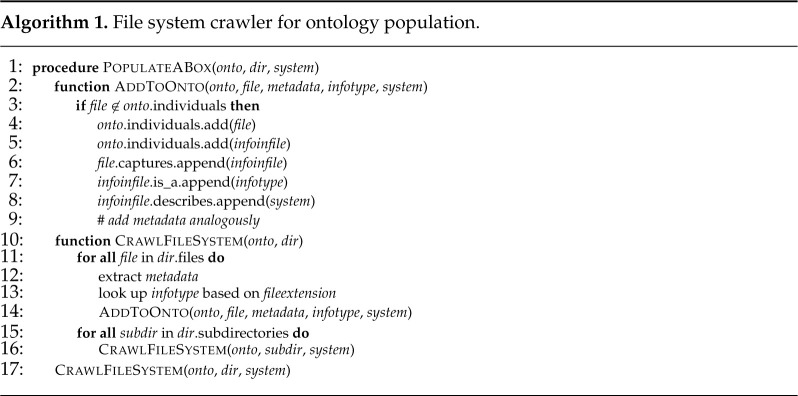


**Figure 3.**
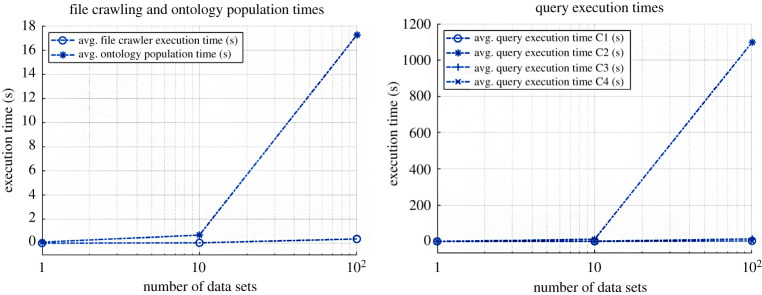
Performance for crawler, ontology creation and query execution.

The approach was evaluated at the example of the data and models available for the extended pick and place unit (xPPU).^3^ The xPPU is a suitable demonstrator, since a wide variety of engineering documents is available, ranging from mechanical CAD models to IEC 61131-3 code to simulations to documentation. This collection of models amounts to 1.20 GB, including text and binary files in various file formats. To assess scalability, we multiplied the available data set by a factor of 10 and 100, respectively, cp. figure 3 (left). The execution times of the queries *(C1–C4)* for information extraction as originally presented by Ocker *et al.* [11] were also acceptable, even without further optimization, cp. figure 3 (right).

The information extracted with this approach cannot only be used to answer certain queries but may also serve as a starting point for automatically creating DTs. Note that, so far, a link between models and the systems they describe is implied via the files’ locations. A detailed semantic analysis of the files has not yet been conducted but would also allow us to automatically link files to the systems they describe.

### (b) Forum mining for ontology extension

The applications, cp. §3., revealed the high potential for hybrid approaches, combining data analytics, expert knowledge and KBSs. In the following, recent research results are presented using NLP to extract expert knowledge out of natural language documents. The extracted information provides the opportunity to develop a KBS. Prior to the results, the initial situation of the use case is discussed. During maintenance of aPSs, several reports are written by the operators and the maintainers. First of all, the operator notifies the maintainer in case of a machine breakdown by describing the symptoms. The maintainer has to diagnose the aPS and take appropriate actions to restore full functionality and restart operation. Besides the existing manuals or maintenance instructions for recurring and known instances, maintainers mostly rely on their experience and expert knowledge. To complete a maintenance job, the maintainer writes a report about the cause of the failure and the maintenance actions. These reports of the operator and the maintainer contain valuable information, which are not used systematically nowadays. However, supporting the maintainer in this experience-based task could accelerate the diagnosis and increase the availability of the aPS significantly. In order to develop a KB, Karray *et al.* 2019 introduced a reference ontology for maintenance (ROMAIN) [65]. Based on BFO [15] a team of experts defined relations and entities for maintenance. A validation was performed on one use case of maintaining an engine of an heavy mobile equipment operator. However, this specific use case cannot cover the whole range of instances. Therefore, the validation is a proof of concept rather than an evaluation, experiencing the limits of the ontology. To overcome this drawback historic maintenance reports can be processed with NLP techniques providing a broad basis for the evaluation and the bottom-up development of an ontology, respectively. In this work, 1,216 posts from a German car maintenance forum are processed in Python using spaCy [62] to extract entities and relations, which should be represented by the maintenance ontology. Typical NLP steps are performed to extract information from unstructured text, cp. Algorithm 2. A pre-trained language model for German is used for part-of-speech (POS) tagging, dependency analysis and lemmatization. Since forum posts oftentimes lack correct grammar and punctuation, a customized sentencizer is used, splitting the sentences at each punctuation and each paragraph. Furthermore, a relevance check is required, eliminating all irrelevant terms included in forum conversations. For this, each term is evaluated based on two additional databases. If a term appears in one of the 2,530 recall forms of the German automobile club’s website (ADAC), the term is considered relevant for car maintenance. If not, it is checked whether the term appears in one of 2548 posts from a cooking forum (chefkoch.de). A term appearing in the cooking forum is considered irrelevant, while a term not appearing in the cooking forum is considered relevant in case of a frequency larger than 1.


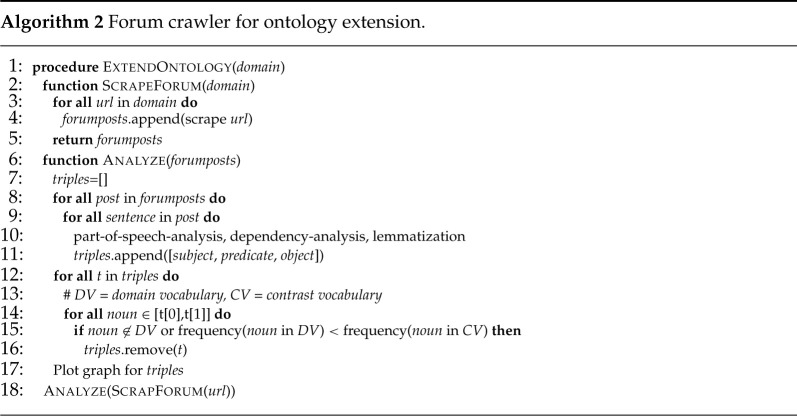


In total, 403,041 words are extracted from the 1216 car maintenance posts yielding 99 667 individual terms. After the relevance check 20 769 relevant terms remain. To reduce the manual effort, the 100 terms with the highest frequency and their five most common relations are further processed to evaluate the reference ontology for maintenance ROMAIN. From 233 term and relation triples, 163 triples can be classified by ROMAIN. The five most frequent ones are shown in table 7. The most frequent triple describes an asset (maintainable item) which suffers from a fault (nonconformity). However, to the best of our knowledge, ROMAIN fails to classify 70 of the extracted triples. One of the most frequent triples deals with the operation mode (cp. table 8). For the diagnosis, it is crucial to distinguish whether a fault occurs under load, e.g. at high speed on a highway, or in idle state, e.g. waiting at a red traffic light. The same applies for aPSs. The data-driven analysis of a maintenance forum revealed the potential of hybrid approaches. The extracted terms and relations showed the deficiencies of a top-down developed ontology. Integrating both approaches provides the possibility to develop and maintain a holistic ontology for maintenance. Similarly, NLP may be applied to automatically extract information from natural language documents created during engineering. Such information could then be represented, e.g. in a formalized information model [11] or in DTs.

**Table 7.** Entities and relations of ROMAIN identified in a German car maintenance forum (idiomatic translations of German terms are added in parentheses).

ROMAIN	OCC	example
Maintainable item, Nonconformity, is BearerOf	33	motor (motor), probleme (problems), haben (have)
Maintainable item, State of Degradation, participatesIn	27	steuerkette (camshaft timing belt), km (km), haben (have)
Asset, Maintainable item, hasPart	23	motor (motor), steuerkette (camshaft timing belt), haben (have)
Maintainable item, Process of Degradation, participatesIn	18	wagen (car), km (km), fahren (drive)
Maintainable item, Function, hasFunction	14	motor (motor), gas (acceleration), geben (increase)

**Table 8.** Categories for entities and relations, which should be added to ROMAIN (idiomatic translations of German terms are added in parentheses).

category	OCC	example triple
cost coverage	13	kosten (costs), BMW (BMW), übernehmen (bear)
operation mode	12	kmh (kilometres per hour), autobahn (highway), fahren (drive)
maintenance provider	8	ölwechsel (oilchange), bmw (bmw), machen (perform)
certificate	3	TÜV (vehicle inspection certificate by the Technical Inspection Agency), wagen (vehicle), bekommen (receive)

## 5. Summary and outlook

By definition, DTs depend on up-to-date and consistent data, information and knowledge. DTs not only require these as inputs but can also be considered models themselves, which can be used for various applications in the context of production systems. This paper provided insights into why the combination of expert knowledge, data analytics and KBSs is essential for production systems in general and the creation of DTs in particular. For this, we answered four research questions. First, we gave an overview, which data, information and knowledge is available throughout the lifecycle of production systems (*RQ1*). Second, we introduced a variety of applications that can benefit from data analysis or KBSs throughout the lifecycle of production systems (*RQ2*). Third, we present an overview of approaches from data analysis and KBSs that have already successfully been applied to production systems (*RQ3*). This selection of applications shows the potential of these technologies but also demonstrates the challenges for state-of-the-art technologies. Finally, we describe the potential for combining data analysis and KBSs in the context of production systems (*RQ4*) and describe two successful feasibility studies that demonstrate how KBS can be created.

Based on the insights gained from the analysis conducted, we suggest several research directions. In general, further research should be conducted to create techniques engineers can use for combining the benefits of expert knowledge, data analytics and KBS. Here, table 5 gives a detailed overview of potential approaches to be researched. Among these challenges, we would like to specifically stress the importance of three aspects. For data analysis, the choice of appropriate algorithms is still a major challenge, while the successful application of KBs would immensely benefit from automated approaches for ontology creation and merging. Finally, appropriate interfaces are key to ensuring the acceptance of novel technologies by engineers and experts. Such interfaces need to be developed both to externalize the engineers’ knowledge and also to provide comprehensible explanations to them.

## Footnotes

1 https://github.com/robert-mieth/information-model-ifac2020, last retrieved July 22, 2021.

2 https://github.com/felixocker/information-model-ifac2020, last retrieved July 22, 2021.

3 https://www.mw.tum.de/ais/forschung/demonstratoren/ppu/, last retrieved July 22, 2021.

## Data accessibility

This article has no additional data.
